# Possible association between high social anxiety level and genetic markers in young adult Internet addicts

**DOI:** 10.1192/j.eurpsy.2022.2107

**Published:** 2022-09-01

**Authors:** A. Trusova, A. Gvozdetckii, T. Merkulova, N. Chuprova, M. Solovieva, S. Grechany, V. Soldatkin, A. Yakovlev, P. Ponizovsky, R. Iluyk, A. Egorov, E. Krupitsky, A. Kibitov

**Affiliations:** 1 Saint Petersburg State University, Department Of Medical Psychology And Psychophysiology, Saint Petersburg, Russian Federation; 2 North-Western State Medical University named after I.I. Mechnikov, Department Of Psychiatry And Addiction, Saint-Petersburg , Russian Federation; 3 Serbsky National Medical Research Center on Psychiatry and Addictions, Laboratory Of Molecular Genetics, Moscow, Russian Federation; 4 Saint Petersburg State Pediatric Medical University, Department Of Psychiatry And Addictology, Saint Petersburg, Russian Federation; 5 Rostov State Medical University, Department Of Psychiatry, Addictology And Medical Psychology, Rostov-on-Don, Russian Federation; 6 Lipetsk Regional Addiction Hospital, Department Of Addictology, Lipetsk, Russian Federation; 7 Serbsky National Medical Research Center on Psychiatry and Addictions, Department Of Therapy Of Mental Disorders Complicated By Addiction Diseases, Moscow, Russian Federation; 8 Bekhterev National Medical Research Center for Psychiatry and Neurology, Department Of Addictology, Saint Petersburg, Russian Federation; 9 Sechenov Institute of Evolutionary Physiology and Biochemistry of the Russian Academy of Sciences, Laboratory Of Neurophysiology And Pathology Of Behavior, St.Petersburg, Russian Federation

**Keywords:** internet addiction, social anxiety, genetic factors

## Abstract

**Introduction:**

Internet addiction (IA) is a rapidly growing disorder especially among adolescents and young adults. Social anxiety is one of the risk factors for IA. Also, genes involved in dopaminergic and serotoninergic systems are among the candidate genes most frequently associated with IA.

**Objectives:**

The study aimed to investigate the association between social anxiety level and genetic markers in young adult Internet addicts.

**Methods:**

IA group included 44 people (Chen/Chinese Internet Addiction Scale (CIAS) score ≥ 65), 75,0% males), the average age 22,0 [18,0;25,0] y.o. (Md [Q1; Q3]). Healthy control group (CIAS score was less 65) included 120 people, (73,3% males), the average age 23,0 [22,0;24,0] y.o. Psychometric measures: Liebowitz Social Anxiety Scale (LSAS). Genetic markers: rs2072450 in *GRIN2A*, rs2832407 in *GRiK-GluR5*, *HUMTH01* in *TH01*(S<9, L>=9 repeats). The impact of genotypes on social anxiety scores was identified using Proportional Odd Logit modeling taking into account group affiliation.

**Results:**

Group of IA reported significantly higher levels in almost all LSAS measures including total score. We found that carriers of the genotypes rs2072450 CC (p=0.004 vs.CA/AA), rs2832407 CC (p=0.023 vs AA), and TH01 SS (p=0.013 vs. LL) scored significantly higher of LSAS total in the IA group. There were no significant differences in the healthy controls group.

**Conclusions:**

The rs2072450(CC) in *GRIN2A*, rs2832407(CC) in *GRiK-GluR5*, and *HUMTH01* in *TH01*(SS) genotypes may be possibly associated with higher social anxiety levels in Internet addicts.

**Disclosure:**

The study was supported by the Russian Foundation for Basic Research (RFBR), project #18-29-22079.

